# Social Determinants of Health Amplify the Association Between Ethnicity and COVID19: A Retrospective-Cohort study

**Published:** 2021-10-27

**Authors:** Nicholas Verdini, Jessica LeClair, Elizabeth Quinn, Amer El-Haddad

**Affiliations:** 1 B.S, Tufts University School of Medicine, MA, USA.; 2 B.S, Department of Biostatistics, Boston University School of Public Health.; 3 M.D., Lynn Community Health Center.

**Keywords:** Social determinants of health, COVID-19, Healthcare disparities

## Abstract

**Background:**

People in racial and ethnic minority groups have been shown to be at increased risk for a variety of diseases, including COVID-19. However, the role that social needs play in this increased risk has not yet been quantified. Investigating these roles can elicit a greater understanding of how social needs influence the manner in which this disease is contracted and spread.

**Methods:**

A retrospective analysis was conducted of 1,969 Lynn Community Health Center patients. Patients that visited the center between February 1st and July 1st, 2020, tested for COVID-19, and screened for social determinants of health (SDOH) risk factors. Demographics were compared between COVID-19 positive and negative patients. Confounding by age on the association between ethnicity and COVID-19 status was evaluated. A stratified analysis was performed to evaluate the effect modification of SDOH on the relationship between race, ethnicity, and COVID-19 status.

**Results:**

Hispanic patients had 2.93 times the odds of a positive COVID-19 test compared to non-Hispanics (95% CI: 2.37 – 3.64, p<0.0001). With at least one SDOH risk factor, Hispanics had 4.71 times the odds of a positive COVID-19 test relative to non-Hispanics (95% CI: 3.10 – 7.14). With no SDOH risk factors, Hispanics had 2.45 times the odds of a positive COVID-19 test relative to non-Hispanics (95% CI: 1.91 – 3.16). No significant associations were found for race.

**Conclusion:**

Ethnicity had a significant impact on COVID-19 status in our population, where the effect of ethnicity on COVID-19 status was amplified for those with SDOH risk factors.

## Introduction

The novel coronavirus, COVID-19, was first reported in Wuhan, China in December of 2019. The COVID-19 outbreak has since been declared a global pandemic, with the first United States case confirmed on January 20th, 2020 in Washington state^1^. Transmission of the virus occurs primarily from person-to person via respiratory droplets produced by coughing, sneezing, or talking.^2^ Within the United States, Massachusetts has been one of the states most severely affected, with one of the highest cumulative incidence rates of COVID-19.^5^ To this end, Massachusetts launched a “Stop the Spread” initiative on July 10th, 2020, to provide free testing, regardless of symptoms, to eight of the most affected towns in the state, many of which are economically disadvantaged. One of the cities included in this initiative was the city of Lynn.^6^ Lynn has a median household income of $56,181, compared to the national median of $68,703.^7^ Additionally, 16.6% of the city population lives in poverty, compared to 10.5% nationally.^7^ To understand why cities such as Lynn are particularly vulnerable to COVID-19, our study aimed to elucidate the influence of race, ethnicity, and social determinants of health on COVID-19 diagnosis.

It is known that essential worker status, congested housing, incarceration, lack of access to healthcare, and diseases like tuberculosis, HIV, and diabetes mellitus increase the risk of COVID-19 infection. ^2–4^ These risk factors disproportionately affect racial and ethnic minority groups.^8^ According to the Center for Disease Control’s (CDC), as of February 28th, 2021, 21% of COVID-19 cases are of Hispanic ethnicity, 12.2% are Black, and 56% are White. However, Black persons account for 18% of the U.S population, Hispanics and Whites account for 13% and 76.3% of the population, respectively.^9^ The CDC also found that racial and ethnic minority groups have four to five times higher rates of hospitalization from COVID-19 compared to non-Hispanic white persons, as well as increased rates of death.^10^ This suggests that minority populations are disproportionately affected by COVID-19. These disparities are likely due to long-standing systemic racism and social inequalities present in both society and the medical system.

Although the CDC has determined a relationship between racial and ethnic minorities with COVID-19 status, that research was limited in that the relationship of these minority groups and COVID-19 status was not evaluated with respect to social determinants of health (SDOH). Social determinants of health are defined by the U.S. Department of Health and Human Services as “the conditions in the environment where people are born, live, learn, work, play, worship, and age that affect a wide range of health, function, and quality-of-life outcomes and risks.” ^11^ The relationship between SDOH and numerous illnesses is well documented.^12–14^ The purpose of this study is to evaluate the relationships between race, ethnicity, social risk factors, and COVID-19 in our population of Lynn Community Health Center (LCHC) patients.

## Materials or patients and methods

### Patient Population

This study was conducted using data compiled from electronic health records of the Lynn Community Health Center (LCHC). Aggregate-level data was collected using the Sheer Dicer software in Epic, an electronic medical record software, and an IRB informed consent waiver was obtained from Lynn Community Health Center and Tufts University School of Medicine. We extracted all LCHC patient medical records that fit the following inclusion criteria: i) the patient visited LCHC between February 1st, 2020, and July 1st, 2020, ii) the patient was tested for COVID-19, and iii) the patient was screened for four social determinants of health (SDOH) risk factors (food access, transportation access, utility status, and housing status). 1,969 LCHC patients met the inclusion criteria and were included in the final analysis sample. All patients meeting this inclusion criteria were included. There were no exclusions or eliminations from this group. Demographics including age group, sex, race, ethnicity, zip code of residence, and SDOH status were also extracted, if available. Note that because we did not require nonmissing demographics as inclusion criteria, some patients in our analysis sample may be missing these demographics. For this reason, while we have a final analysis sample size of 1,969, this is not necessarily the number included in every analysis utilizing demographic data. Collection of human data was in accordance with guidelines within the Declaration of Helsinki.

### SDOH Screening

We utilized Epic data from an SDOH questionnaire provided by Community Care Cooperative, a MassHealth Accountable Care Organization, provided in supplemental materials. The questionnaire consisted of eight questions (one of which was the date) regarding social environment, including: housing status and adequacy, food insecurity, lack of access to transportation, risk of utilities being shut off, and job status. This questionnaire was derived from the 26 question Accountable Health Communities Health-Related Social Needs Screening Tool. The Centers for Medicare and Medicaid constructed the screening tool with a panel of national experts and review of existing screening instruments.^17–18^ The questionnaire was shortened to eight questions by a coordination between Community Care Cooperative, Massachusetts Medical-Legal Partnership of Boston, and Lynn Community Health Center in order to make this an appropriate over-the-phone screening tool. The screening was conducted after patients were tested for COVID-19 and was documented in their electronic medical records. Patients were flagged as at risk if they selected any option on any question that was not “I am not sure,” “None of the above,” “No,” or “Never true.”

### Statistical Methods

The analysis sample included 1,969 LCHC patients who met the inclusion criteria. Distributions of patient demographics were descriptively compared between COVID-19 positive and negative patients. To assess any differences in these baseline demographics, chi-square tests and logistic regressions were performed.

In addition to these bivariate tests of association, the intricate relationships between ethnicity, social determinants of health, and COVID-19 status were further investigated by evaluating possible confounding and effect modification. Namely, the extent of confounding by age on the association between ethnicity and COVID-19 status was evaluated by assessing whether the percent change in Cochran Mantel-Haenszel (CMH) odds ratio estimates was more than 10% from the unadjusted odds ratios. Additionally, to evaluate the possibility of effect modification by SDOH on the relationship between ethnicity and COVID-19 status, a stratified analysis was performed. Odds ratios describing the association were calculated for those with at least one SDOH risk factor and those with no SDOH. To assess the presence of effect modification, the Breslow-Day Test for Homogeneity of the Odds Ratios was performed. A two-sided significance level of 5% was used to determine statistical significance in all analyses. SAS 9.4 was used to perform all analyses.

## Results

### Patient Demographics

Our analysis sample included 1,969 patients from LCHC (Table 1). Of these 1,969 patients, there were 969 (49.21%) patients with a positive COVID-19 test result and 1000 (50.79%) patients with a negative result. For both COVID-19 positive and negative patients, patients in the 18–44 years old age group were the largest cohort. The majority of patients self-identified as White, of which 443 were COVID-19 positive and 512 were COVID-19 negative. 442 patients self-identified as Black, of which 198 were COVID-19 positive and 244 were negative. Self-identifying Hispanic patients made up the greatest number of patients in both positive and negative patients, of which 792 were COVID-19 positive and 619 were COVID-19 negative. Most patients in the study were negative for at least one SDOH risk factor, of which 701 were COVID-19 positive and 739 were COVID negative. The zip code 01902 made up the overwhelming majority for the place of residence for this patient group. Finally, female patients made up 61.71% of patients in our study (Table 1).

### Association of patient demographics with COVlD-19 status

Of the demographics collected in this study, age (p<0.0001), ethnicity (p<0.0001), zip code (p=0.0102), and sex (p<0.0001) were significantly associated with COVID-19 infection (Table 1). Race (=0.5789) and the presence of at least one SDOH (p=0.4357) were not statistically significant (Table 1). In our sample, Hispanic patients had 2.93 times the odds of testing positive for COVID-19, compared to non-Hispanics (95% CI: 2.37 – 3.64) (Table 2a). Although the presence of at least one SDOH risk factor was not in itself significant with COVID-19 (Table 1), the possibility of effect modification of the association between ethnicity and COVID-19 status by SDOH risk factors was evaluated. This was done via an SDOH-stratified analysis (Table 2b). When at least one SDOH risk factor is present, Hispanics have 4.71 times the odds of testing positive for COVID-19 relative to non-Hispanics (95% CI: 3.10 – 7.14). Contrastingly, when there are no SDOH risk factors present, Hispanics have 2.45 times the odds of testing positive for COVID-19 relative to non-Hispanics (95% CI: 1.91 – 3.16). Given that these odds ratios are significantly different as evidenced by the Breslow-Day test (Table 2b, p-value = 0.0085), there is evidence of effect modification by SDOH risk factors. Of note, a similar analysis first testing effect modification of SDOH, then confounding, was performed for the association between race and COVID status. However, we did not have evidence of effect modification by SDOH, nor confounding by SDOH (Supplemental Table 3, Breslow-Day p-value = 0.9075, CMH p-value = 0.5783).

Given that age was significantly associated with COVID-19 status (Table 1, p-value <0.0001) and that age and ethnicity were also significantly associated (Supplemental Table 4, p-value <0.0001), the extent of confounding by age on the association between ethnicity and COVID status was also evaluated. Ultimately, adjusting for age only changed the odds ratio estimate by 4.1%. This suggested confounding by age on this association was minuscule, and hence the unadjusted results were appropriate (Table 2a). Because SDOH risk factors were demonstrated to be an effect modifier on the relationship of ethnicity and COVID-19 status, it would have been ideal to evaluate the extent of confounding by age on this more complex association. However, due to low cell counts produced when splitting the data into multiple strata, this adjustment was not possible.

## Discussion

The COVID-19 pandemic has impacted the daily lives of all people. However, this pandemic has not affected all people equally. In our patient cohort, ethnicity had a significant impact on COVID-19 status, where being of Hispanic ethnicity (versus not being of Hispanic ethnicity) alone was a significant risk factor for COVID-19. This is consistent with the CDC’s national finding of higher rates of COVID-19 among the Hispanic population.^10^

### Results Interpretation

This disparity in COVID-19 among ethnic groups in our cohort was amplified by the presence of SDOH risk factors. While the presence of an SDOH risk factor alone was not significantly associated with COVID-19 status, it proved to be a significant effect modifier on the ethnicity and COVID-19 relationship. In other words, our results show that those who are Hispanic are at an increased risk of COVID-19 infection and those who are Hispanic and have a SDOH risk factor are at an even greater risk. This increase in COVID-19 risk for Hispanics with a SDOH risk factor is disproportionate compared to non-Hispanics in our population. These findings may be due to a variety of systemic factors and inequities in social determinants of health that put racial and ethnic minorities at increased risk for disease. The Hispanic population has been shown to experience discrimination, inadequate healthcare access and utilization, inequities in education access, wealth gaps, and increased congested housing, all of which increase the risk of contracting COVID-19.^19–26^ In Lynn, MA where 42.8% of the population is Hispanic^7^, addressing these discrepancies in health is of great importance in order to control COVID-19 and future health crises.

While this data does not describe why SDOH status and ethnicity cause such a significant change in COVID-19 status, it provides tangible evidence that these disparities do exist and that they affect health. This highlights the importance of recognizing, studying, and making changes to the inequalities that lead to these social disparities. SDOH have also proven to lead to disproportionate adverse health outcomes in many other instances, like premature mortality, mental illness, congenital anomalies, Type 2 Diabetes, and bacterial infections.^27–30^ Additionally, COVID-19 is not the first pandemic where SDOH have played a role in enlarging health disparities amongst minorities and those of lower socioeconomic status. For instance, the United States HIV epidemic has shown a greater overall illness burden amongst those at the lowest levels of socioeconomic status,^32^ primarily of minority ethnicity and race. Even in the 1918 influenza pandemic, research has shown that those living in Chicago neighborhoods with higher illiteracy had increased risk of influenza mortality.^33^

Additionally, age was significantly associated with COVID-19 positivity. This is a well-known finding and many hypotheses have been published as to why age influences susceptibility to COVID-19. According to CDC data, age distribution for COVID-19 cases follows a bell-curve relationship, with those between the ages of 18 and 64 making up the greatest number of positive cases.^34^ While there are many biological reasons as to why this may be the case, i.e., ACE2 receptor density amongst different age groups, there are also proposed social reasons for this. Long-term care facilities for elderly, as well as daycare and public schools were amongst the first to institute COVID-19 restrictions. On March13th, 2020, the Centers for Medicare & Medicaid Services issued a lockdown order, banning everyone but essential personnel from entering nursing homes.^35^ On March 16^th^, the Commonwealth of Massachusetts ordered the closure of all public and private elementary and secondary schools.^36^ This decrease in contact with other persons in these two age groups prevents transmission of COVID-19.^37–39^

### Limitations

Although this study highlighted the important relationship that SDOH plays in the COVID-19 pandemic in our cohort, it has limitations. In our patient cohort, we did not find the same result that the CDC and other publications had concerning race and COVID-19 status. This may be due to limitations within the questionnaire. Limitations include the lack of SDOH questions concerning specific housing conditions (e.g., congestion, ability to social distance), essential worker status, income, and medical insurance status. These limitations may have resulted in patients not being included in the study that had SDOH risk factors. These SDOH risk factors that were not included have been shown to be significantly related to COVID-19 cases.^24–26^ In particular, essential worker status is an important metric that was not assessed by this questionnaire. Essential worker status has been shown to be associated with greater COVID-19 infection and mortality. This association is not free from disparity. Research has shown that Non-Hispanic Blacks disproportionately occupy essential-worker positions compared with Non-Hispanic Whites.^26^ By not assessing essential-worker status, it is possible that Black patients who did have this SDOH were not included in the study.

The SDOH Questionnaire is also a limitation of the study in that the reliability and validity of this tool have yet to be investigated. While the tool was constructed by an experienced panel based on commonly used or evidence-based questions, the questionnaire has not been tested as a unit and therefore data on reliability and validity is not available. Additionally, patients may not feel comfortable disclosing these personal parts of their social environment and this will cause an under detection of patients with SDOH risk. Our patient population was limited to those who were tested at Lynn Community Health Center and therefore, these results may not necessarily be generalizable to other populations. Finally, we did not evaluate whether there was an association between sex and ethnicity in our sample, which if significant, would have indicated potential confounding by sex. Acknowledging these limitations, we nonetheless report an association between ethnicity, SDOH, and COVID-19 status.

## Conclusion

Investigating the underlying causes for the stark ethnic differences in COVID-19 infection rates can lead to a greater understanding of the virus spread and may help control it. Further research should be conducted, with a focus on a larger spectrum of demographics and social determinants of health to develop a greater understanding of the sociodemographic disparities in this pandemic and other health disparities. Additionally, measures should be taken to proactively record SDOH risk status as patients are tested for COVID-19 in order to provide a greater perspective of SDOH effects on acquisition and spread of the virus. Increased quantitative data linking SDOH and health outcomes would be influential in changing health policy. A lack of data on health equity outcomes, as well as methods that work to reduce disparities in said outcomes has been cited as a major obstacle to policy change.^40^ The number of studies that evaluating public policy and its impact on health equity continue to rise, but the number is still relatively small. A few states in the U.S., one example being Oregon, are utilizing systems incentivizing providers based on equity performance. It will be important to evaluate the effects this has on health equity outcomes.^41^

An increased focus on addressing social disparities in healthcare can aid in preventing a similar pattern in future health crises and decrease the gap of adverse health outcomes amongst minority populations in the United States. Additionally, a standardized method for healthcare systems to collect SDOH data and the impacts of programs and policy designed to address disparities in health outcomes must be implemented in order to make advances and prevent further studies needing to cite lack of data as a barrier to improvement.

## Supplementary Material

1

## Figures and Tables

**Table 1.** Association Between Demographics and COVID-19 Status

**Variable**	COVID Test Result		P-value
	Positive (N=969)	Negative (N=1,000)	
**Age**			
**Under 18**	63 (6.50%)	33 (3.30%)	<0.0001
**18–44**	575 (59.34%)	499 (49.90%)	
**45–64**	274 (28.28%)	382 (38.20%)	
**65+**	57 (5.88%)	86 (8.60%)	
**Race**			0.5789
**Black**	198 (30.89%)	244 (32.28%)	
**White**	443 (69.11%)	512 (67.72%)	
**Ethnicity**			<0.0001
**Hispanic**	792 (83.46%)	619 (63.23%)	
**Non-Hispanic**	157 (16.54%)	360 (36.77%)	
**At least one SDOH Risk Factor**			
**Positive**	268 (27.66%)	261 (26.10%)	0.4357
**Negative**	701 (72.34%)	739 (73.90%)	
**Zip Code**			
**01901**	22 (2.48%)	44 (5.54%)	0.0102
**01902**	537 (60.54%)	473 (59.57%)	
**01904**	72 (8.12%)	69 (8.69%)	
**01905**	256 (28.86%)	208 (26.20%)	
**Sex**			
**Male**	422 (43.55%)	332 (33.20%)	<0.0001
**Female**	547 (56.45%)	668 (66.80%)	

**Table 2a:** Association between Ethnicity and COVID-19, Before and After Adjusting for Age*

	COVID Test Result	
Ethnicity	Positive (N=949)	Negative (N=979)
**Hispanic**	792 (83.46%)	619 (63.2370)
**Non-Hispanic**	157 (16.54%)	360 (36.77%)
**Unadjusted Odds Ratio (95% CI): 2.93 (2.37, 3.64)**		
**Age-Adjusted Odds Ratio (95% CI): 2.81 (2.26, 3.49)**		

* While out study size was 1,969, due to missing data, this analysis sample only contained 1,928.

**Table 2b:** Association Between Ethnicity and COVID-19 Status Stratified by SDOH Risk Factor

At least one SDOH Risk Factor		
Breslow Day Test p-value: 0.0085		
	COVID Test Result	
Ethnicity	Positive (N=260)	Negative (N=258)
**Hispanic**	220 (84.62%)	139 (53.88%)
**Non-Hispanic**	40(15.38%)	119 (46.12%)
Odds ratio (95% CI): 4.71 (3.10, 7.14)		
No SDOH Risk Factors		
	COVID Test Result	
Ethnicity	Positive (N=689)	Negative (N=721)
**Hispanic**	572 (83.02%)	480 (66.57%)
**Non-Hispanic**	117 (16.98%)	241 (33.43%)
**Odds ratio (95% CI): 2.45 (1.91, 3.16)**		

* While our study sample size was 1,969, due to missing data, this analysis sample only contained 1,928 patients.

## References

[1] Holshue ML, DeBolt C, Lindquist S, Lofy KH, Wesman I, Bruce H, First Case of 2019 Novel Coronavirus in the United States. N Engt J Med 2020; 382(10):929–36. 10.1056/NEJMoa2001191 PMC7092802 32004427

[2] Centers for Disease Control and Prevention. COVID-19 Overview and Infection Prevention and control Priorities in non-US Healthcare Settings Available from: https://www.cdc.gov/coronavirus/2019-ncov/hcp/non-us-settings/overview/index.html. Last updated February 26th, 2021; cited April 28th, 2021

[3] Rashedi J, Mandavi Poor B, Asgharzadeh V, Pourostadi M, Samadi Kafil H, Vegari A, Tayebi-Khosroshahi H, Asgharzadeh M. Risk Factors for COVID-19. Infez Med 2020 Dec 1;28(4):469–474. 33257620

[4] Song H, McKenna R, Chen AT, David G, & Smith-McLallen A (2021). The impact of the non-essential business closure policy on Covid-19 infection rates. International Journal of Health Economics and management, 1–40. 10.1007/s10754-021-09302-9 PMC8012742 33792808

[5] Massachusetts Department of Public Health. COVID-19 Response Reporting Available from: https://www.mass.gov/info-details/covid-l9-response-reporting. Last updated: Daily; cited April 28th, 2021.

[6] Massachusetts Department of Public Health. Baker-Polio administration announces new initiatives to stop spread of covid-19 Available from: https://www.mass.gov/news/baker-polito-administration-announces-new-initiatives-to-stop-spread-of-covid-19. Last updated: August 7th, 2020; cited April 28th, 2021.

[7] Bureau USC. QuickFacts: Lynn, Massachusetts.

[8] Turner-Musa J, Ajayi O, Kemp L. Examining Social Determinants of Health, Stigma, and COVID-19 Disparities. Healthcare (Basel) 2020; 8(2). 10.3390/healthcare8020168 PMC7349778 32545647

[9] Centers for Disease Control and Prevention.. CDC COVID Data Tracker Available from: https://covid.cdc.gov/covid-data-tracker/#demographics. Last updated: August 24th, 2021; cited April 12th, 2021.

[10] Centers for Disease Control and Prevention. COVID-19 Hospitalizations and Death by Race/Ethnicity Available from: https://www.cdc.gov/coronavirus/2019-ncov/covid-data/investigations-discovery/hospitalization-death-by-race-ethnicity.html. Last updated: July 16th, 2021; cited: April 6th, 2021.

[11] U.S. Department of Health and Human Services. Healthy People 2030 Available from: https://health.gov/healthypeople/obiectives-and-data/social-determinants-health. Last updated: unavailable; cited August 25th, 2021.

[12] Song R, Hall HI, Harrison KM, Sharpe TT, Lin LS, Dean HD. Identifying the impact of social determinants of health on disease rates using correlation analysis of area-based summary information. Public Health Rep 2011; 126 Suppl 3: 70–80. 10.1177/00333549111260S312 PMC3150132 21836740

[13] Cockerham WC, Hamby BW, Oates GR. The Social Determinants of Chronic Disease. Am J Prev Med 2017; 52(1 S1): S5–S12. 10.1016/j.amepre.2016.09.010 PMC5328595 27989293

[14] Butler-Jones D, Wong T. Infectious disease, social determinants and the need for intersectoral action. Can Commun Dis Rep 2016; 42(Suppl 1): S118–S20. 10.14745/ccdr.v42is1a04 PMC5868637 29770035

[15] Singh GK, Daus GP, Allender M, Ramey CT, Martin EK, Perry C, Social Determinants of Health in the United States: Addressing Major Health Inequality Trends for the Nation, 1935–2016. Int J MCH AIDS 2017; 6(2): 139–64. 10.21106/ijma.236 PMC5777389 29367890

[16] Lynn Community Health Center. Lynn Community Health Center General Information Available from: https://www.ichcnet.org/lynn-community-health-center-general-information. Last updated: unavailable; cited May 6th, 2021.

[17] Centers for Medicaid and Medicare Serves.. The accountable health communities health-related social needs screening tool Available from: https://innovation.cms.gov/files/worksheets/ahcm-screeningtool.pdf. Last updated: unavailable; cited April 29th, 2021.

[18] Billoux A, Verlander K, Anthony S, Alley D Standardized screening for health-related social needs in clinical settings: The accountable health communities screening tool. NAM Perspectives 2017.

[19] In: Smedley BD, Stith AY, Nelson AR, editors. Unequal Treatment: Confronting Racial and Ethnic Disparities in Health Care Washington (DC); 2003P. eng 25032386

[20] Promotion OoDPaH. Social Determinants of Health 2020.

[21] Statistics USBoL. Labor force characteristics by race and ethnicity 2018.

[22] Cox R, Seva R, Benjamin H, Suzanne W Measuring population estimates of housing insecurity in the United States: A comprehensive approach. CESR - Schaeffer Working Paper 2017.

[23] Centers for Disease Contol.. Communities, Schools, Workplaces, & Events: 2020. Available from: https://www.cdc.gov/coronavirus/2019-ncov/community/health-equity/race-ethnicity.html?CDC_AA_refVal=https%3A%2F%2Fwww.cdc.gov%2Fcoronavirus%2F2201-ncov%2Fneed-extra-precautions%2Fracial-ethnic-minorities.html#fn17. Last updated: April 19th, 2021; cited: April 8th, 2021.

[24] Macias Gil R, Marcelin JR, Zuniga-Bianco B, Marquez C, MathewT, Piggott DA. COVID-19 Pandemic: Disparate Health Impact on the Hispanic/Latinx Population in the United States. J Infect Dis 2020; 222(10): 1592–5. 10.1093/infdis/jiaa474 PMC7454709 32729903

[25] Centers for Disease Control. Considerations for owners and operators of multifamily housing including populations at increased risk for complications from covid-19 Available from: https://www.cdc.gov/coronavirus/2019-ncov/community/multifamily-housing.html. Last updated: May 5th, 2021; cited: May 6th, 2021.

[26] Rogers TN, Rogers CR, VanSant-Webb E, Gu LY, Yan B, Qeadan F. Racial Disparities in COVID-19 Mortality Among Essential Workers in the United States. World Med Health Policy 2020. 10.1002/wmh3.358 PMC7436547 32837779

[27] Stringhini S, Carmeli C, Jokela M, Avendano M, Muennig P, Guida F, Socioeconomic status and the 25 x 25 risk factors as determinants of premature mortality: a multicohort study and meta-analysis of 1.7 million men and women. Lancet 2017; 389(10075): 1229–37. 10.1016/S0140-6736(16)32380-7 PMC5368415 28159391

[28] Vukojevic M, Zovko A, Talic I, Tanovic M, Resic B, Vrdoljak I, Parental Socioeconomic Status as a Predictor of Physical and Mental Health Outcomes in Children - Literature Review. Acta Clin Croat 2017; 56(4): 742–8. 10.20471/acc.2017.56.04.23 29590731

[29] Vrijheid M, Dolk H, Stone D, Abramsky L, Alberman E, Scott JE. Socioeconomic inequalities in risk of congenital anomaly. Arch Dis Child 2000; 82(5): 349–52. 10.1136/adc.82.5.349 PMC1718336 10799420

[30] Walker RJ, Smalls BL, Campbell JA, Strom Williams JL, Egede LE. Impact of social determinants of health on outcomes for type 2 diabetes: a systematic review. Endocrine 2014; 47(1): 29–48. 10.1007/s12020-014-0195-0 PMC7029167 24532079

[31] Coffey PM, Ralph AP, Krause VL. The role of social determinants of health in the risk and prevention of group A streptococcal infection, acute rheumatic fever and rheumatic heart disease: A systematic review. PLoS Negl Trap Dis 2018; 12(6): 60006577. 10.1371/journal.pntd.0006577 PMC6016946 29897915

[32] Pellowski JA, Kalichman SC, Matthews KA, Adler N. A pandemic of the poor: social disadvantage and the U.S. HIV epidemic. Am Psychol. 2013; 68(4): 197–209. 10.1037/a0032694 PMC3700367 23688088

[33] Grantz KH, Rane MS, Salje H, Glass GE, Schachterle SE, Cummings DA. Disparities in influenza mortality and transmission related to sociodemographic factors within Chicago in the pandemic of 1918. Proc Natl Acad Sci USA 2016; 113(48): 13839–44. 10.1073/pnas.1612838113 PMC5137773 27872284

[34] Centers for Disease Control. Demographic Trends of COVID-19 cases and deaths in the US reported to CDC Available from: https://covid.cdc.gov/covid-data-tracker/#demographics. Last updated: August 24th, 2021; cited August 25th, 2021.

[35] Centers for Medicare and Medicaid Services. Guidance for Infection Control and Prevention of Coronavirus Disease 2019 (COVID-19) in Nursing Homes Available from: https://www.cms.gov/files/document/qso-20-14-nh-revised.pdf. Last updated: March 13th, 2021; cited August 25th, 2021.

[36] Commonwealth of Massachusetts. Order extending the temporary closure of all public and private elementary and secondary schools Available from: https://www.mass.gov/doc/april-21-2020-school-closure-extension-order/download. Last updated: April 21st, 2020; cited August 25th, 2021.

[37] Auger KA, Shah SS, Richardson T, Association Between Statewide School Closure and COVID-19 Incidence and Mortality in the US. JAMA 2020;324(9):859–870. doi:10.1001/jama.2020.14348 PMC7391181 32745200

[38] Brauner JM, Mindermann S, Sharma M, Johnston D, Salvatier J, Gavenciak T, … & Kulveit J (2021). Inferring the effectiveness of government interventions against COVID-19. Science, 371(6531). 10.1126/science.abd9338 PMC7877495 33323424

[39] Yang B, Huang AT, Garcia-Carreras B, Hart WE, Staid A, Hitchings MD & Cummings DA (2021). Effect of specific non-pharmaceutical intervention policies on SARS-C0V-2 transmission in the counties of the United States. Nature communications, 12(1), 1–10. 10.1038/s41467-021-23865-8 PMC8195990 34117244

[40] Lee J, Schram A, Riley E, Harris P, Baum F, Fisher M, Freeman T, & Friel S (2018). Addressing Health Equity Through Action on the Social Determinants of Health: A Global Review of Policy Outcome Evaluation Methods. International journal of health policy and management, 7(7), 581–592. 10.15171/ijhpm.2018.04 PMC6037500 29996578

[41] Oregon leverages Medicaid to address social determinants of health and health equity. Center for Health Systems Effectiveness, Oregon Health & Science University; 2021.

