# Supplementary material for: Social Determinants of Health Amplify the Association Between Ethnicity and COVID19: A Retrospective-Cohort study
Source: Int J Med Stud. Author manuscript; Available in PMC 2021 Dec 23. (PMC8699061)
Supplement: 1 [file NIHMS1754063-supplement-1.pdf]

Supplemental Table 3: Association Between Race and COVID-19 Status Stratified by SDOH Risk Factor

At least one SDOH Risk Factor		
Breslow Day Test p-value: 0.9075		
	COVID Test Result	
Race	Positive	Negative
Black	48 (30.77%)	63 (31.66%)
White	108 (69.23%)	136 (68.34%)
Odds ratio (95%CI): 0.96 (0.61,1.51)		

No SDOH Risk Factors		
	COVID Test Result	
Race	Positive	Negative
Black	150 (30.93%)	181 (32.50%)
White	335 (69.07%)	376 (67.50%)
Odds ratio (95%CI): 0.93 (0.72,1.21)		

Supplemental Table 4: Evaluating the Association Between Age and Ethnicity to Evaluate Age as a Potential Confounder

	Ethnicity		
**Age**	**Hispanic**	**Non-Hispanic**	**p-value**
Under 18	76 (5.39%)	16 (3.09%)	<0.0001
18-44	803 (56.91%)	246 (47.58%)	
45-64	448 (31.75%)	199 (38.49%)	
65+	84 (5.95%)	56 (10.83%)	

Supplementary Materials: Social Determinants of Health Questionnaire

ENGLISH

Name ______________________________

DOB ________________________________

Medical Record # ___________________

Directions: Please fill out all the questions, whether you are answering for yourself or for a child, so that your care team has the most complete information to care for you.

1. Today's Date: ____/____/______

2. What is your housing situation today?
   - ❑ I do not have housing (staying with others, in a hotel, in a shelter, living outside on the street, on a beach, in a car, or in a park)
   - ❑ I have housing today, but I am worried about losing housing in the future
   - ❑ I have housing
   - ❑ I am not sure

3. Think about the place you live. Do you have problems with any of the following? (Check all that apply)
   - ❑ Pests such as bugs, ants, or mice
   - ❑ Mold
   - ❑ Lead paint or pipes
   - ❑ Inadequate heat
   - ❑ Oven or stove not working
   - ❑ No or not working smoke detectors
   - ❑ Water leaks
   - ❑ None of the above
   - ❑ I am not sure

4. Within the past 12 months, you worried that your food would run out before you got money to buy more.
   - ❑ Often true
   - ❑ Sometimes true
   - ❑ Never true

5. Within the past 12 months, the food you bought just didn't last and you didn't have enough money to get more.
   - ❑ Often true
   - ❑ Sometimes true
   - ❑ Never true

English 06/19

STAFF ONLY

Completed:
❑ In-person ❑ By mail
❑ By phone ❑ By Email
❑ Other

6. In the past 12 months, has lack of transportation kept you from medical appointments, meetings, work or from getting things needed for daily living? (Check all that apply)
   - ❑ Yes, it has kept me from medical appointments or getting medications
   - ❑ Yes, it has kept me from non-medical meetings, appointments, work, or getting things that I need
   - ❑ No
   - ❑ I am not sure

7. In the past 12 months has the electric, gas, oil, or water company threatened to shut off services in your home?
   - ❑ Yes
   - ❑ No
   - ❑ Already shut off
   - ❑ I am not sure

8. Do you want help finding or keeping work or a job?
   - ❑ Yes, help finding work
   - ❑ Yes, help keeping work
   - ❑ I do not need or want help
   - ❑ I am not sure

COMMUNITY CARE COOPERATIVE
